# Supplementary material for: Establishing a counter-empathy processing model: evidence from functional magnetic resonance imaging
Source: Soc Cogn Affect Neurosci. 2021 Aug 31;17(3):273–89. doi: 10.1093/scan/nsab097 (PMC8881639; doi:10.1093/scan/nsab097)
Supplement: nsab097_Supp [file nsab097_supp.zip › supplementary.docx]

**Establishing a** **Counter-Empathy** **Processing Model: Evidence from Functional Magnetic Resonance Imaging**

Jing Jie^1,2,3,4^, Min Fan^1,3,4^, Yong Yang^1,3,4,5^, Pinchao Luo^1,3,4^, Yijing Wang^1,3,4^, Junjiao Li^1,3,4,6^, Wei Chen^1,3,4^, Mengdi Zhuang^1,3,4^, Xifu Zheng*^1,3,4^

1.School of Psychology, South China Normal University, Guangzhou, China

2.School of Biomedical Engineering, Hainan University, Haikou, China

3.Center for Studies of Psychological Application, South China Normal University, Guangzhou, China

4.Guangdong Key Laboratory of Mental Health and Cognitive Science, South China Normal University, Guangzhou, China

5. School of Educational Science, Xinyang Normal University, Xinyang, China

6. School of Teacher Education, Guangdong University of Education, Guangzhou, China

**Address for correspondence:** Xifu Zheng, School of Psychology, South China Normal University, Guangzhou, 510631, China; Tel: 86-20-85216386. Fax: 86-20-85216412. E-mail: zhengxf@scnu.edu.cn

**Abstract:**

Counter-empathy significantly affects people’s social lives. Previous evidence indicates that the degree of counter-empathy can be either strong or weak. Strong counter-empathy easily occurs when empathizers are prejudiced against the targets of empathy (e.g., prejudice against outgroup members) and activates brain regions that are opposite to those activated by empathy. Weak counter-empathy may have different neural processing paths from strong one, but its underlying neural mechanisms remain unclear. In this work, we used an unfair distribution paradigm, which can reduce participants’ prejudice against persons empathized with, and functional magnetic resonance imaging (fMRI) to explore the neural mechanisms underlying counter-empathy. Here, empathy and counter-empathy shared a common neural mechanism, induced by unfair distribution, in the right middle temporal gyrus. Counter-empathy activated distinct brain regions that differed from those of empathic responses in different situations. The functions of these brain regions, which included the middle frontal, middle temporal and left medial superior gyri, were similar and mostly related to emotional regulation and cognitive processing. Here, we propose a process model of counter-empathy, involving two processing paths according to whether or not prejudice exists. This study has theoretical significance and broadens our understanding of the cognitive neural mechanisms underlying empathy and counter-empathy.

Keywords: counter-empathy; empathy; process modeling; fMRI; neural mechanism; inequality

**1. Introduction**

Empathy is an important contributor to successful social interaction and a key motivator for altruistic behavior. Therefore, researchers are interested in empathy and its underlying cognitive neural mechanisms. Counter-empathy, corresponding to empathy, significantly affects human social lives, but its underlying neural processing remain unclear. Counter-empathy refers to emotional reactions that are incongruent, or even at odds, with the emotional states of others (Yamada et al., 2011). Counter-empathy, discovered by early researchers (Stotland et al., 1971; Aderman & Unterberger, 1977; Englis et al., 1982), is a common everyday psychological phenomenon. Some examples of counter-empathic phenomena are if one becomes jealous rather than happy when a colleague one considers annoying receives a promotion or if one is unhappy when a sports team one dislikes scores. Another example is when psychopathic killers do not feel their victim’s pain and may even feel pleasure. With the rise of cognitive neuroscience research, scholars have begun paying attention to the conditions triggering counter-empathy and its underlying cognitive neural mechanisms. And some valuable findings are obtained. The results of these studies further support that counter-empathy is the opposite of empathy or that counter-empathy is inconsistent with empathy.

Several fMRI studies have shown that counter-empathy activates brain regions that are opposite to those activated by empathy (Dvash et al., 2010; Hein et al., 2010; Singer et al., 2006; Takahashi et al., 2009). Singer et al. (2006) found that men exhibited counter-empathic responses when they observed a person who had acted unfairly receiving pain. This effect was accompanied by increased activation in reward-related areas (e.g., the nucleus accumbens), which correlated with an expressed desire for revenge. Takahashi et al. (2009) found that when the target person’s possession was superior and self-relevant, participants were more envious and showed stronger activation in the anterior cingulate cortex (ACC, a brain area related to pain empathy). Their subsequent study revealed that when misfortunes happened to envied persons, participants felt stronger schadenfreude and showed stronger activation in the striatum, a reward-related brain area. Hein et al. (2010) found that activation of the anterior insula (a brain area related to pain empathy) and associated self-reports of empathic concern best predicted whether participants helped an ingroup member when seeing them suffer. Conversely, activation of the nucleus accumbens (a reward-related brain area) and the degree of negative evaluation of another best predicted whether participants did not help an outgroup member.

Other studies have shown that counter-empathy manifests as the weakening of empathy; that is, one’s emotional responses are inconsistent with another’s emotional responses (Chiao & Mathur, 2010; Decety et al., 2010; Feng et al., 2016; Itagaki & Katayama, 2008; Luo, Pang et al., 2018; Yamada et al., 2011). An event-related potential (ERP) technology study found that counter-empathy occurred in the late stages of empathic responses to others’ economic payoffs. The authors of that study argued that counter-empathy induced by unfair distribution was not a complete disregard for others’ interests, but an emotional reaction accounting for others’ interests while focusing on one’s own interests. Their study showed that the relationship between counter-empathy and empathy may be a transformation relationship and not the opposite (Jie, Luo, Zhuang, Li et al., 2019). Fan et al. (2021) found that social exclusion downregulates empathic responses in the late stages of empathic responses and that this modulation is attenuated gradually.

We drew two conclusions from these studies. First, the degree of counter-empathy can be either strong or weak. Strong counter-empathy and weak counter-empathy may have different processing paths. Second, to our knowledge, strong counter-empathy easily occurs when empathizers are prejudiced against the targets of empathy. Prejudice is an irrational, negative, or hostile attitude toward a person or members of another group. Examples include prejudice against outgroup members (e.g., fans of different soccer teams, Hein et al., 2010), people one does not like (Singer et al., 2006), or people one envies (Takahashi et al., 2009). That is, before experiencing counter-empathy, individuals either experience prejudice towards outgroup members (Han, 2018; Hein et al., 2010), or they dislike the targets of empathy for various reasons (Singer et al., 2006; Takahashi et al., 2009). Because there are two types of counter-empathy, many unanswered questions remain about its neural mechanisms.

Thus, we further explored the potential neural mechanisms of counter-empathy, and we propose two hypotheses. First, if people reduce their preconceived prejudices against others, it will weaken their counter-empathic responses, which manifests inconsistently with the emotional reactions of others. Second, counter-empathy may involve more complex psychological mechanisms than those of empathy. According to previous ERP studies, one reason for this is that counter-empathy induced by some contexts occurs at the late stages of empathic responses, exhibiting a transformation from empathy to counter-empathy (Fan et al.,2021; Jie, Luo, Zhuang, Li et al., 2019). Another reason is that counter-empathic facial expressions are more complex than empathic facial expressions. An electromyography study indicated that stronger reactions occurred under the “schadenfreude” condition than under the joy condition (Boecker et al., 2015). Another study found that even if 6-year-old children had to pay money, they still wanted to watch the punishment of an antisocial agent and showed signs of schadenfreude (Mendes et al., 2018). However, they displayed more frequent smiles coupled with frowns during the punishment of an antisocial agent than during the punishment of a prosocial agent, thus showing a complex emotion (Mendes et al., 2018).

In the current study, we adopted a similar paradigm to that of Jie, Luo, Zhuang, Li et al. (2019) because this paradigm can reduce participants’ prejudice against persons empathized with. The participants had never met their partners, of the same race (ingroup), before participating in the experiment and the flag-matching task (Materials and Methods section) made the participants and their partners more like collaborators, thus alleviating participants’ preconceived biases towards the targets of empathy. Second, in this experimental paradigm, participants saw their partners’ emotional facial expressions, which helped induce empathic responses. According to the perception-action model of empathy, a perceived emotional state automatically activates the respective emotional state in the observer, along with the associated autonomic and somatic responses (Preston & de Waal, 2002). People can often determine the emotional states of others by reading their facial expressions (Balconi & Pozzoli 2007, 2009). Several neuroimaging studies have provided empirical evidence for this model, showing overlapping neural responses during the direct experiences and the passive observation of emotions and sensations (Wicker et al., 2003). Previous studies have also used facial emotional expressions to induce empathic responses (de Greck, Scheidt et al., 2012; de Greck Shi, et al., 2012; Lamm et al., 2007; Schulte-Rüther et al., 2008). Third, the current paradigm asked participants to evaluate the degree of unpleasantness they experienced for their partner’s outcome. Participants perceived their partner’s facial expressions and provided emotional feedback representing that perceived emotion. This helped improve the participants’ attention to the other’s emotions and easily induced empathy. Fourth, the current paradigm asked participants to imagine their partner’s feelings as much as possible. Previous studies have found that when people imagine others’ feelings, they can easily experience similar feelings, which enables them to empathize with others (Decety & Grezes, 2006). Similarly, if participants failed to produce consistent emotions when perceiving others’ facial expressions, it indicated that they produced counter-empathy. Some researchers have adopted a similar paradigm to induce counter-empathy (Jie, Luo, Zhuang, Fan et al., 2019; Jie, Luo, Zhuang, Li et al., 2019; Yamada et al., 2011).

In conclusion, the current study used fMRI to explore the cognitive neural mechanism of counter-empathy induced by unfair distribution and its relationship with cognitive neural processing of empathy when no preconceived prejudice exists against others. Exploring the neural mechanisms behind counter-empathy induced by unfair distribution and understanding how this state of inner imbalance is generated and regulated can broaden the research on the cognitive neural mechanisms of empathy and counter-empathy. It can also provide scientific guidance for clinical intervention treatment and behavior correction methods and help regulate individual negative emotional reactions, enhance individual happiness, and maintain social stability.

**2. Material and Methods**

***2.1. Participants***

We performed power and sample size estimations using Superpower (Lakens & Caldwell, 2021) in the online Shiny apps (see https://arcstats.io/shiny/anova-power/). Based on the simulations results, 20 participants were enough to detect a significant effect. For a detailed overview of this simulations, please see our supplementary file. Then we recruited thirty-eight participants (19 women, 19 men; mean age, 20.58 ± 1.90 years) for payment. All participants were right-handed, had normal or corrected-to-normal vision, and no history of neurological or psychological disorders. The Research Ethics Review Board of South China Normal University approved the study protocol (approval number 148). All participants provided written informed consent according to the Declaration of Helsinki before the experiment. The study was divided into two experiments: an empathy-induced experiment and a counter-empathy-induced experiment. All participants met the criteria for MR scanning. In the two experiments, three and two participants, respectively, were excluded for excessive motion during the scan (>3 mm in any ordinal direction or a 3° pitch, roll, or yawn). The empathy and counter-empathy experiments included 35 and 36 valid participants, respectively, in the one-sample t-test and 33 valid participants in the paired-sample t-test in the final fMRI data analyses.

***2.2. Materials***

***2.2.1 Flag-matching task***

Images used in the experiment were downloaded from the online Flag Picture Gallery: 192 images of flags were selected and divided into 96 pairs. In a pilot study, we asked 20 individuals to choose one image from each image pair that they thought most people would prefer. To ensure that the payoff distribution task in the formal experiment had credibility, if one image was selected by more than 80% of the participants, it meant that the participants and their partners had a high probability of choosing the same flag; therefore, this outcome was designated as a “correct choice” in the computer program in the subsequent formal experiment. If both flags were selected with equal probability (in the 40%–60% range), it indicated that the probability of the participants and their partners choosing the same national flag would be relatively low, and both flags were designated as “incorrect choices” in the subsequent formal experiment. Forty-eight image pairs were used in this study.

***2.2.2 Expression feedback***

We engaged a male volunteer and a female volunteer as partners (confederates) in the experiment. Three still photos of a male partner and a female partner, clearly expressing a discernible frown, neutral expression, or smile, in response to a disadvantageous inequality (DI) distribution, a fair (FA) distribution, or an advantageous inequality (AI) distribution for their partner were shot against a white wall background and evaluated in advance by 28 healthy adults. The valence and arousal of these expressions were rated on a 9-point scale, with higher scores indicating higher levels of valence or arousal. The Greenhouse-Geisser correction was applied for the violation of the sphericity assumption in ANOVA where appropriate, and the Bonferroni correction was used for multiple comparisons. The valence scores differed significantly between expression types (smile: 6.46 ± 0.14; neutral: 3.70 ± 0.21; frown: 1.88 ± 0.15). There were significant differences between each of the three expressions (*ps* < 0.001). The arousal degree also significantly differed between the expression types (smile: 5.59 ± 0.23; neutral: 3.20 ± 0.17; frown: 5.07 ± 0.29). The results of multiple comparisons showed that the arousal degrees for smile and frown were significantly higher than that of the neutral expression (*p* < 0.001), but the difference between smile and frown was not significant. This suggested that pictures of these three facial expressions can induce negative, neutral, and positive emotions. The brightness, size, contrast, and color settings of the pictures were unified with photo editing software.

***2.3. Procedure***

The experiment used a within-subjects design of 2 (conditions: empathy/counter-empathy) × 2 (feedback: correct/incorrect) × 3 (level of fairness to others: DI, FA, or AI).

Before the scanning session, participants were introduced to their partner. Participants were asked to stand against the wall and have their pictures taken with the three expressions (frown, neutral, and smile) using a digital camera. Similarly, three pictures of the partners’ three expressions were taken in the presence of the participant. Then, both the participant and confederate were given a comprehensive description of the tasks they would perform. The participants signed the research consent form at the MRI lab and completed the Screening Form of the Brain Imaging Center of South China Normal University. To ensure that the participants understood the experimental process, they were tested on the content of the experimental process and could participate in the formal experiment only if they correctly answered 100% of the questions. The entire experiment consisted of two parts: the empathy experiment and the counter-empathy experiment.

***2.3.1 Empathy experiment***

Participants watched their partner playing a flag-matching game with a computer (Fig. 1). The reason why the partners didn’t complete the task with the third-party persons is that it could refrain the participants from empathy for the third-party persons, which would affect their empathic responses to their partner.

Participants were told that if the computer and their partner chose the same image, a “√” would appear on the screen, and then the computer and their partner would share a 10 yuan bonus. There were three bonus distribution types for the partners. If the partner’s frown was presented, it meant a DI distribution (the partner got 0 yuan; the computer got 10 yuan). The partner’s neutral expression meant an FA distribution (the partner and the computer each got 5 yuan). The partner’s smile meant an AI distribution (the partner got 10 yuan; the computer got 0 yuan). If the computer and the partner chose different images, an “×” appeared on the screen, and the computer and the partner incurred a 10 yuan fine together. There were three fine distributions for the partners. The partner’s frown represented a DI distribution (the partner paid 10 yuan; the computer paid 0 yuan). The partner’s neutral expression meant an FA distribution (the partner and computer each paid 5 yuan). The partner’s smile meant an AI distribution (the partner paid 0 yuan; the computer paid 10 yuan). Participants were required to try their best to imagine their partner’s situation and experience their partner’s feelings during the experiment. At the end of each trial, the participants completed a rating scale to measure the degree of unpleasantness they experienced for their partner’s outcome, which was divided into two ratings. Participants’ subjective feelings were assessed via valence ratings of their affective state. The feeling was unpleasant, neutral, or pleasant, corresponding to keys 1, 2, and 3, respectively. They were also asked the degree of formal feelings they experienced, which was rated on a 4-point scale. Larger numbers meant stronger feelings, corresponding to keys 1, 2, 3, and 4. If participants chose “neutral” in the previous interface, then the second interface would not appear. The combination of these two interfaces was similar to a 9-point subjective rating, ranging from very unpleasant to very pleasant.

***2.3.2*** ***Counter-empathy experiment***

The counter-empathy experiment involved a similar manipulation to that of the empathy experiment (Fig. 1). The difference was that the participants and their partners played a flag-matching game together. The participants were asked to choose one flag from the pair by pressing keys 1 or 2 within 3 seconds. They were then shown three still shots, clearly expressing a discernible frown, neutral expression or smile, in response to a DI, FA, or AI distribution to the partner, respectively.

After explaining the experimental procedure, participants were told that their partner would participate in the same experiment in another room, and the partner would see the photos of the participants’ three facial expressions (frown, neutral, or smile, in response to a DI, FA, or AI distribution, respectively). The bonus in the participant fee was derived from the average bonus earned by the participant in the correctly matched trials.

The task was equally divided into four blocks (two empathy blocks and two counter-empathy blocks) with 36 trials each. All experiment conditions within a block were randomized across participants. Half of the participants completed the empathy task and then the counter-empathy task, while the other half did the opposite. The entire experiment consisted of 144 trials, with 12 trials per condition.

[INSERT FIGURE 1 HERE]

***2.4 fMRI data acquisition***

All fMRI data were acquired on a 3.0 T MR scanner (Trio Tim, Siemens Medical Systems, Erlangen, Germany) with a 12-channel head coil at the Brain Imaging Center of South China Normal University. The fMRI data were acquired using an echo-planar imaging (EPI) sequence with the following parameters: repetition time = 2000 msec, echo time = 30 msec, flip angle = 90°, matrix = 64 × 64, field of view = 204 × 204 mm^2^, thickness/gap = 3.5/0.8 mm, voxel size = 3.5 × 3.5 × 3.5 mm^3^ and 32 axial slices covering the whole brain. During the scan, participants were instructed to remain still in the scanner throughout the entire experiment and avoid any movement. Participants with excessive head motions were excluded from the data analysis (see section 2.5).

***2.5 fMRI data analysis***

Neuroimaging data were preprocessed and analyzed using Dpabi (Yan, Wang, Zuo, & Zang, 2016). Slice timing and head motion correction were performed first. Data for three and two participants were excluded because of excessive motion during the scan (>3 mm in any ordinal direction or a 3° pitch, roll, or yawn) in the empathy and counter-empathy experiments, respectively. The preprocessed data were normalized to the standard Montreal Neurological Institute (MNI) space by EPI template and resampled to 3-mm isotropic voxels. Smoothing was conducted with a 6-mm full-width-half-maximum Gaussian kernel to suppress noise and effects due to residual differences in functional and gyral anatomy during intersubject averaging.

Preprocessing statistical analysis was performed using the general linear model (Friston et al., 1994) in SPM12 (Statistical Parametric Mapping, [www.fil.ion.ucl.ac.uk/spm/](http://www.fil.ion.ucl.ac.uk/spm/)) and Matlab_R2013b to establish participants’ voxel-wise activation during expression feedback epochs (see the red box in Figure 1). These regressors were convolved with a canonical hemodynamic response function. Activated voxels in each experimental context were identified using an event-related statistical model. First-level model estimation of the empathy and counter-empathy experiment included six conditions: 2 (feedback: correct/incorrect) × 3 (level of fairness to others: frown, neutral, or smile, in response to a DI, FA, or AI distribution, respectively). On the second level, simple t-tests were performed for “empathy contrast” and “counter-empathy contrast”. Specifics for empathy and counter-empathy were analyzed by entering the respective first-level contrast images into a factorial design. Excluding the participants with excessive head motion, the single-sample t-tests of the empathy and counter-empathy experiment analyzed 36 and 35 participants’ data, respectively. The specific contrasts “empathy > counter-empathy” and “counter-empathy > empathy” were then inclusively masked for significant voxels of the respective simple contrast. Excluding the participants with excessive head motion, the paired-sample t-test included 33 participants’ data. All contrasts were thresholded at a cluster level of *p* < 0.05 for the familywise error (FWE) corrected level and *p* < 0.001 for the voxel level. Anatomical labeling was performed automatically using XjView (http://www.alivelearn.net/xjview). We used BrainNet Viewer (Xia, Wang, & He, 2013) to view the significant fMRI results. The Automatic Anatomic Labeling Atlas was used to parcellate the cerebral cortex into 90 predefined cortical regions (Tzourio-Mazoyer et al., 2002).

**3. Results**

***3.1 Behavioral data***

Each person conducted 12 subjective pleasure ratings under each condition. The Greenhouse-Geisser correction was applied for the violation of the sphericity assumption in ANOVA where appropriate, and the Bonferroni correction was used for multiple comparisons. Table 1 shows the mean scores and standard deviations of the subjective pleasure ratings in each context. Repeated-measures analysis of variance was conducted for the behavioral data, with condition (empathy/counter-empathy), feedback (correct/incorrect), and fairness (DI/FA/AI) as the three independent factors. The interaction effect of the three variables was significant: *F*_(2,74)_ = 12.67, *p* < 0.001, *η^2^_p_* = 0.26.

[INSERT TABLE 1 HERE]

Further simple effect analysis revealed that for the counter-empathy condition, the mean subjective pleasure score for DI was significantly higher than that for the empathy condition, regardless of whether the feedback was correct (*F*_(1, 37)_ = 158.01, *p* < 0.001, *η^2^_p_* = 0.81) or incorrect (*F*_(1, 37)_ = 50.32, *p* < 0.001, *η^2^_p_* = 0.58). In the counter-empathy condition, the mean subjective pleasure score for AI was significantly lower than that for the empathy condition, regardless of whether the feedback was correct (*F*_(1,37)_ = 40.03, *p* < 0.001, *η^2^_p_* = 0.52) or incorrect (*F*_(1,37)_ = 77.74, *p* < 0.001, *η^2^_p_* = 0.68). This showed that compared with the empathy conditions, when the participants were under counter-empathy conditions, they were happier about their partner’s DI distribution and less happy about their partner’s AI distribution. For correct feedback, participants’ subjective pleasure to their partner’s FA distribution in the counter-empathy condition was significantly higher than that for the empathy condition (*F*_(1,37)_ = 7.30, *p* < 0.05, *η^2^_p_* = 0.17). For incorrect feedback, participants’ subjective pleasure to their partner’s FA distribution in the counter-empathy condition was significantly lower than that for the empathy condition (*F*_(1, 37)_ = 27.18, *p* < 0.001, *η^2^_p_* = 0.42; Figure 2).

[INSERT FIGURE 2 HERE]

Under the empathy condition, regardless of whether the feedback was correct, the main effect of fairness was significant, (*F_correct_*_(2,36)_ = 31.58, *p* < 0.001, *η^2^_p_* = 0.64; *F_incorrect_*_(2,36)_ = 24.01, *p* < 0.001, *η^2^_p_* = 0.57). Participants rated themselves as feeling happier with the AI distribution than with the FA (*t_correct_*_(37)_ = 4.94, *p* < 0.001; *t_incorrect_*_(37)_ = 3.20, *p* < 0.01) or DI (*t_correct_*_(37)_ = 6.70, *p* < 0.001; *t_incorrect_*_(37)_ = 5.59, *p* < 0.001) distributions, regardless of the feedback. They also felt happier with the FA than with the DI distribution (*t_correct_*_(37)_ = 7.80, *p* < 0.001; *t_incorrect_*_(37)_ = 6.86, *p* < 0.001). For the counter-empathy condition, regardless of the feedback, the main effect of fairness was significant (*F_correct_*_(2,36)_ = 27.61, *p* < 0.001, *η^2^_p_* = 0.61; *F_incorrect_*_(2,36)_ = 17.94, *p* < 0.001, *η^2^_p_* = 0.50). Participants rated themselves as feeling less happy with the AI distribution than with the FA (*t_correct_*_(37)_ = -6.04, *p* < 0.001; *t_incorrect_*_(37)_ = −5.51, *p* < 0.01) and DI (*t_correct_*_(37)_ = −7.41, *p* < 0.001; *t_incorrect_*_(37)_ = −5.98, *p* < 0.01) distributions, regardless of the feedback. They also felt less happy with the FA than with the DI distribution: *t_correct_*_(37)_ = −6.90, *p* < 0.001; *t_incorrect_*_(37)_ = −4.88, *p* < 0.001; Figure 3.

[INSERT FIGURE 3 HERE]

### *3.2 fMRI results*

***3.2.1 Common brain areas involved in empathy and counter-empathy***

Compared with baseline (the normal time), most conditions in the empathy and counter-empathy experiments (for details, see Tables 2 and 3, and Figures 4 and 5) significantly activated participants’ right middle temporal gyrus (rMTG). Additionally, all conditions in the counter-empathy experiments significantly activated participants’ left middle temporal gyrus (lMTG) and right medial superior frontal gyrus (rMESFG; *p* < 0.001, uncorrected at the voxel level, *p* < 0.05 FWE-corrected at the cluster level). Tables 2 and 3 and Figures 4 and 5 show the activation results for the brain areas under each condition.

[INSERT TABLE 2 HERE]

[INSERT FIGURE 4 HERE]

In the empathy experiment, the right superior occipital gyrus (rSOG) and the rMTG were significantly activated under correct feedback with the AI distribution (C_AI) to the partner. The bilateral MTG, left middle occipital gyrus (lMOG), rSOG, right inferior frontal gyrus, triangular part (rIFGtriang), and right precuneus were significantly activated when the feedback was incorrect with a DI distribution (I_DI) to the partner. The rSOG, rMTG, left angular gyrus (lAG), and right precuneus were significantly activated when the feedback was incorrect with an FA distribution (I_FA) to the partner. The rMTG and lAG were significantly activated when the feedback was incorrect with an AI distribution (I_AI) to the partner.

In the counter-empathy experiment, the bilateral MTG, rMESFG, lAG, and left middle frontal gyrus (lMFG) were significantly activated when the feedback was correct with a DI distribution to the partner (C_DI). The bilateral MTG, bilateral MFG, left inferior frontal gyrus, triangular part (lIFGtriang), rMESFG, left inferior parietal lobule (lIPL), supramarginal and angular gyri, and right precuneus were significantly activated with correct feedback and an FA distribution to the partner (C_FA). The bilateral MTG, bilateral MFG, right inferior frontal gyrus, orbital part (rORBinf), lIFGtriang, rMESFG, and right precuneus were significantly activated with correct feedback and an AI distribution to the partner (C_AI). The bilateral MTG, rMESFG, right middle occipital gyrus (rMOG), and right precuneus were significantly activated with incorrect feedback and a DI distribution to the partner (I_DI). The bilateral MTG, bilateral MFG, lIFGtriang, lAG, right angular gyrus (rAG), rMESFG, right precuneus, and left superior frontal gyrus (lSFG) were significantly activated with incorrect feedback and an FA distribution to the partner (I_FA). The bilateral MTG, rMESFG, lAG, right precuneus, and lMFG were significantly activated with incorrect feedback and an AI distribution to the partner (I_AI).

[INSERT TABLE 3 HERE]

[INSERT FIGURE 5 HERE]

***3.2.2 Specific brain areas involved in empathy and counter-empathy***

*3.2.2.1 C_DI distribution to partner*

During the facial expression phase, empathy minus counter-empathy activated the right precentral gyrus (rPreCG). Counter-empathy minus empathy activated the bilateral MFG, rAG, and lIPL (*p* < 0.001 uncorrected at the voxel level, *p* < 0.05 FWE-corrected at the cluster level; Table 4, Figures 6A and B).

[INSERT TABLE 4 HERE]

[INSERT FIGURE 6 HERE]

*3.2.2.2 I_DI distribution to partner*

During the facial expression phase, empathy minus counter-empathy activated the rPreCG. Counter-empathy minus empathy activated the bilateral ORBinf, rMTG, rMESFG, lIPL, and rAG (*p* < 0.001 uncorrected at the voxel level, *p* < 0.05 FWE-corrected at the cluster level; Table 5, Figures 7A and B).

[INSERT TABLE 5 HERE]

[INSERT FIGURE 7 HERE]

*3.2.2.3 C_AI distribution to partner*

During the facial expression phase, empathy minus counter-empathy showed no significant differences. Counter-empathy minus empathy activated the lIFG, triangular, bilateral MTG, bilateral AG, and lMESFG (*p* < 0.001 uncorrected at the voxel level, *p* < 0.05 FWE-corrected at the cluster level; Table 6, Figure 8).

[INSERT TABLE 6 HERE]

[INSERT FIGURE 8 HERE]

*3.2.2.4 I_AI distribution to partner*

During the facial expression phase, empathy minus counter-empathy activated the rPreCG. No significant differences were found for counter-empathy minus empathy (*p* < 0.001 at the voxel level, *p* < 0.05 FWE-corrected at the cluster level; Table 7, Figure 9).

[INSERT TABLE 7 HERE]

[INSERT FIGURE 9 HERE]

***3.2.3 Specific brain areas involved in counter-empathy for different levels of fairness***

Comparing the brain activation for counter-empathy under unfair conditions (i.e., a confirmed money loss), DI minus AI activated the lORBinf, right fusiform gyrus, bilateral inferior occipital gyrus (IOG), left thalamus, and left postcentral gyrus (lPoCG) only after incorrect feedback during the facial expression phase. No significant differences were found in AI minus DI (*p* < 0.001 at the voxel level, *p* < 0.05 FWE-corrected at the cluster level; Table 8, Figure 10).

[INSERT TABLE 8 HERE]

[INSERT FIGURE 10 HERE]

I_DI minus C_DI activated the bilateral MTG, rORBinf, right parahippocampal gyrus, lIOG, rMESFG, left middle cingulate gyrus, right precuneus, and right supplementary motor area. No significant differences were found between I_AI and C_AI (*p* < 0.001 uncorrected at the voxel level, *p* < 0.05 FWE-corrected at the cluster level; Table 9 and Figure 11).

[INSERT TABLE 9 HERE]

[INSERT FIGURE 11 HERE]

**4. Discussion**

The results of the self-report measures showed that when self-interest was involved, the unfair distribution led to counter-empathy. The fMRI results indicated that empathy and counter-empathy have both common (e.g., the right middle temporal gyrus) and distinct brain regions activated under different conditions of unfairness to others. Thus, empathy and counter-empathy are not complete opposites. These results demonstrate that if participants are not prejudiced against the targets of empathy, they will not experience strong counter-empathy.

**4.1 Empathy and counter-empathy may not be completely opposed**

The results confirmed that empathy and counter-empathy did not completely oppose each other when no prejudice existed. Empathy and counter-empathy had common activated brain regions (e.g., the right middle temporal gyrus).

As the core region of emotion generation and processing, the middle temporal gyrus (Critchley et al., 2000; Seehausen et al., 2014) participated in processing emotional facial expressions (Vuilleumier et al., 2003), cognitive empathy (Carrington & Bailey, 2009; Völlm et al., 2006), and theory of mind (Hein & Singer, 2008; Kandylaki et al., 2015). One study found that compared with the control stimuli, pain-related exclamations elicited increased activation in the superior and middle temporal gyri, suggesting that the middle temporal gyrus is involved in empathy (Lang et al., 2011). Additionally, greater neural activation occurred for ingroup than for outgroup members in a shared emotional facial expression production and perception network (including the middle temporal gyrus), indicating more neural resonance (mirroring) for ingroup emotional facial expressions. Thus, empathic responses towards ingroup members were stronger than those towards outgroup members (Krautheim et al., 2019). Together with these studies, the current results regarding activation of the middle temporal gyrus during empathy and counter-empathy indicated that empathy and counter-empathy for unfair distributions to others had similar stages of emotional processing, and these similar stages are likely to occur in the early stages of empathy.

Some researchers have proposed two possible systems for empathy: a basic emotional contagion system and a more advanced cognitive perspective-taking system (de Waal, 2007; Shamay-Tsoory et al., 2009). The basic emotional contagion system is thought to support our ability to empathize emotionally (“I feel what you feel”), a process by which sensory emotional information is automatically and unconsciously transmitted between individuals (Shamay-Tsoory et al., 2009). In other words, emotional facial expression processing is the basis of the emotional contagion. Therefore, activation of the middle temporal gyrus may be related to facial expression processing and the emotional contagion, which occurs in the early stages of empathy. These results supported previous ERP research results (Jie, Luo, Zhuang, Fan et al., 2019; Jie, Luo, Zhuang, Li et al., 2019) in which the authors found that although participants were more concerned for their own outcomes than for others’ benefits when self-interest was involved, their empathic responses toward their coplayers were reduced only in the late stages of empathic responses.

Notably, the same brain regions may be activated because both empathy and counter-empathy have similar facial expression processing. However, as mentioned above, processing of emotional facial expressions is closely related to the emotional contagion, and the participants are required to experience their partner's feelings as much as possible. Therefore, simply processing emotional facial expressions without experiencing emotions is difficult.

Empathy and counter-empathy each had their own distinct brain regions. Empathy generally and significantly activated the right precentral gyrus. Previous studies revealed that the precentral gyrus was related to affective empathy (Hooker et al., 2010), cognitive empathy (Seehausen et al., 2014), and emotional recognition (Adolphs et al., 2000). Moreover, the precentral gyrus was the key neural mechanism involved in the “mirror system” of emotional expressions (Carr et al., 2003; Pfeifer et al., 2008). According to the perception-action model of empathy (Preston & de Waal, 2002), perception of a target’s state (e.g., facial expressions and emotional body language) automatically activates the observer’s own representation of that state, which then triggers autonomic and somatic responses. Observers who mimic the experience of the target generate empathy. In current study, empathy activated the right precentral gyrus more significantly than counter-empathy did. Thus, we hypothesized that the neural response of counter-empathy may inhibit the mirror nervous system’s automatic shared representation for others’ facial expressions, thus weakening the empathic response. Although both the right precentral and middle temporal gyri are involved in automatic emotional arousal, they may play different roles in this arousal. The precentral gyrus may be more closely related to mirror-image imitation, while the middle temporal gyrus is more closely related to facial expression processing.

Counter-empathy generally and significantly activated several brain regions, including the right angular gyrus and the left inferior parietal, in addition to activating regions specific to different distributions. The angular gyrus participated in a variety of complex cognitive processes. Specifically, it emerged as a cross-modal hub that combined and integrated converging multisensory information to comprehend and make sense of events, manipulate mental representations, solve familiar problems, and reorient attention to relevant information (Seghier, 2013). Current findings suggested that the IPL may be a relatively critical brain region for counter-empathy under conditions of disadvantageous inequality to others. The IPL has been implicated in social cognition and emotional control and may comprise “hubs” or “switches” in emotional-conflict processing. The IPL prioritizes negative rather than positive cues (Rohr et al., 2016); thus, we inferred that when personal interests are involved, because the IPL prioritizes processing negative information, seeing others’ frowns (disadvantageous inequality distribution to others) would make it easier to activate the IPL. Subsequently, participants would consider that this unfavorable result for their partner may benefit themselves, consequently generating positive emotions. Based on current research and previous results, we speculated that counter-empathy had a more complex cognitive process than that of empathy. When self-interest conflicted with others’ interests and the distribution results were unfavorable to others, the participants had stronger inner conflicts, indicating that the participants were concerned about their partner’s economic losses.

**4.2 Functional commonalities of activated brain regions during counter-empathy for different unfair distributions**

Compared with empathy, although counter-empathy has different brain regions specific to different distributions to others, the functions of these brain regions are similar and are mostly related to emotional regulation and cognitive processing (e.g., the MFG, MTG, MESFG, rIFG, orbital part, AG, and IFGtriang).

The MFG is related to emotional reappraisal (Kim et al., 2013) and attention reorienting (Japee et al., 2015), both of which are emotional regulation strategies (Gross, 1998). Previous studies have found that emotional reappraisal (compared with maintaining one’s emotional response) produced greater activation in the bilateral inferior/middle/superior frontal gyri and temporal gyrus (Kim et al., 2013). The IFG, SFG, and temporal gyrus were all significantly activated here, showing that counter-empathy has more emotional regulation than empathy has. The medial superior frontal gyrus belongs to the medial prefrontal cortex and is activated when participants attribute mental states to others (i.e., mentalizing or theory of mind; Gallagher et al., 2000). Increased neural activity in the MESFG (the rostral part of the supplementary motor area) is also related to attention shift (Nagahama et al., 1999). The angular gyrus has been implicated in attention (Chambers et al., 2004) and emotional regulation (Kohn et al., 2014). The results of these studies also suggest that counter-empathy requires more cognitive processing than empathy does.

We also considered that in the empathy task, participants were simply observers in the flag-matching task; however, in the counter-empathy task, they were active participants. This may cause some confusion in the experimental results in that counter-empathy had a more complex cognitive process than empathy did. However, based on the brain activation and our experimental design, it is unlikely that counter-empathy activated more complex brain regions than the empathy tasks did because of the complexity of the counter-empathy tasks. First, if the difference in brain activation between counter-empathy and empathy was only due to the complexity of the counter-empathy tasks, then under all corresponding conditions, counter-empathy should activate similar and more complex brain areas than empathy should, especially when partners have the same facial expressions. However, this was not the case. For example, although both had disadvantageous inequality distributions to others (seeing the partner’s frown), the brain regions significantly activated by counter-empathy minus empathy differed between the conditions of correct pairing (i.e., no bonus) and incorrect pairing (i.e., a confirmed money loss).

Second, throughout the experiment, participants were asked to experience others’ emotions as much as possible. This experimental design improved participants’ attention to others’ facial expressions and enhanced their internal motivation for concern for the other. Moreover, at the end of each trial, participants were asked to complete the subjective pleasure evaluation, which further increased their motivation to carefully observe their partners’ facial expressions. Therefore, even in the empathy task, participants paid attention to the feelings of others, thus reducing the possibility of their being more involved in the counter-empathy task. Additionally, there was a 1500–2500 ms blank screen before the facial expression to reduce the influence of key selection on the previous screen.

Third, and most importantly, previous studies using facial expressions to trigger empathy (de Greck, Scheidt et al., 2012; de Greck, Shi et al., 2012; Lamm et al., 2007; Schulte-Rüther et al., 2008) and counter-empathy (Jie, Luo, Zhuang, Fan et al., 2019; Jie, Luo, Zhuang, Li et al., 2019; Yamada et al., 2011) have yielded significant results, which demonstrated the validity and feasibility of this research paradigm. Ideally, it would be better to create a situation in which participants are equally active (or passive) in the flag-matching task and manipulate whether participants are motivated to empathize or counter-empathize with their partners. We expect subsequent research to further improve this research paradigm.

Interestingly, counter-empathy more significantly activated the bilateral orbital IFG than empathy did only when others lost money after incorrect matching. However, for correct matching, when participants gained money but their partner did not, although it was a disadvantageous inequality to the partner, no significant activation difference occurred in the orbital IFG. The orbitofrontal cortex is thought to be important for processing rewards (Mainen & Kepecs, 2009) and encodes the current reward representation values accessible to predictive cues (Gottfried et al., 2003; Rolls, 2000). These results showed that when participants’ partners lost money but the participants did not, the participants felt happy, thus activating the orbitofrontal cortex. Furthermore, it showed that participants were happier when they did not lose money than they were when they gained money. This is consistent with the theory of loss aversion (Tversky & Kahneman, 1991). We then wondered whether people were dissatisfied only with personal money losses, or were they also dissatisfied with others’ money losses. Do people only care about their own interests, or do they also care about the others’ interests? We further compared the brain activation during counter-empathy to the partners under disadvantageous inequality and advantageous inequality distributions.

We further analyzed the brain regions that were significantly activated for others’ money loss compared with those when others lost no money (no penalty for an incorrect match or no bonus for a correct match). These brain areas had three main functions. The first involved facial expression recognition and empathy and included the fusiform gyrus (Kanwisher et al., 1997), inferior occipital gyrus (Haxby et al., 2002), postcentral gyrus (Borich et al., 2015), middle cingulate gyrus (Feng et al., 2016; Goubert et al., 2005; Singer et al., 2004), middle temporal gyrus (Carrington & Bailey, 2009; Völlm et al., 2006; Vuilleumier et al., 2003), precuneus (Kandylaki et al., 2015; Seehausen et al., 2014), and parahippocampal gyrus (Borich et al., 2015). The second was the orbital IFG, which involved the schadenfreude in counter-empathy (Breiter et al., 2001; Gottfried et al., 2003; Li et al., 2015; Rolls, 2000). The third involved cognitive processing and emotional regulation and included the parahippocampal gyrus (Borich et al., 2015), medial prefrontal cortex (Buckner et al., 2008; Gao et al., 2018; Perry et al., 2012), middle cingulate gyrus, supplementary motor area (Kohn et al., 2014), and thalamus (Der Werf et al., 2001). This reflected the complexity of brain activity during counter-empathy for others’ money losses.

When their partners lost money, participants’ empathy-associated brain regions were activated more significantly than during other conditions, suggesting that individuals were strongly aware that the other person’s frown was painful and generated unpleasant emotions. Participants then considered their own interests and knew that this meant a favorable outcome for themselves. Thus, the orbitofrontal cortex, a brain area associated with reward, was activated more significantly than it was for other conditions. In this case, when the partner lost money, participants had stronger inner emotional conflicts, which manifested as stronger initial induced empathic responses and stronger subsequent counter-empathy, thus more significantly activating the brain areas related to emotional regulation. That is, participants were concerned not only with their own interests, but with others’ interests, and felt bitter about others’ losses, even if it meant a good outcome for themselves, showing a tendency toward “loss aversion”.

**4.3 Counter-Empathy Processing Model**

Based on current and previous research findings, we proposed that counter-empathy may have two processing paths (Figure 12). First, a path without prejudice against others consists of the following. (1) Affective arousal occurs when emotional cues automatically induce people’s empathic responses. (2) Cognitive evaluation and emotional regulation rely on the awareness of self and others and occur when people distinguish between self and others’ interests, produce cognitive empathy, pay attention to self-interest, and cognitively reappraise their emotions. This is the key stage in inducing counter-empathy. (3) Self-emotion generation occurs when people produce self-emotion after cognitive evaluation. (4) Emotional conflict occurs when self-generated emotions conflict with those induced by others, representing the complexity of counter-empathy. (5) Emotional regulation occurs when people adopt emotional regulation strategies to help generate a flexible and appropriate response. The second path occurs when people are prejudiced against others. This regulates the perception of emotional cues through top-down processing and induces self-emotion (automatic counter-empathy) and empathic responses towards others. This self-emotional state weakens empathic responses. Finally, people adopt emotional regulation strategies to help generate flexible and appropriate responses. This model can reasonably explain the mechanisms of counter-empathy and provide theoretical support for discussing this psychological phenomenon.

[INSERT FIGURE 12 HERE]

**4.4 Limitations and future directions**

This study expands the previous research on neural mechanisms of empathy and suggests a processing model of counter-empathy, thus providing new insight into the relationship between empathy and counter-empathy. However, the study had some limitations. First, we only recruited younger people with physical and mental health, but previous studies have shown that people with high anxiety have a slowed disengagement of attention to threatening stimuli (Fox et al., 2001; Yiend et al., 2001). Another study found that state anxiety inhibited empathic responses from the early emotional-sharing stage to the late cognitive-evaluation stage (Luo, Zhuang et al., 2018). Thus, individuals with high anxiety are more likely to immerse themselves in negative emotions and have difficulty producing appropriate counter-empathic responses at the right time. Additionally, personality traits can also affect counter-empathic responses. For example, individuals’ levels of neuroticism were positively correlated with negative mental health and negatively correlated with positive mental health (Tian et al., 2019). Such individuals may be more prone to perceive negative information for the same conditions. Future studies should expand the scope of research participants.

Second, the current study focused on the neural mechanism of state counter-empathy. A previous study using the Interpersonal Reactivity Index scale found that a callous/unemotional (C/U) dimension was related to deficits in empathy, and C/U traits were associated with an increased focus on the positive aspects of aggression and a decreased focus on the negative aspects of hostile acts (Pardini et al., 2003). Therefore, are some people more prone to counter-empathy? If so, they may also be more likely to have a sense of competition with others and be prone to aggressive behaviors. Using a measurement tool for trait counter-empathy could enable identifying people prone to counter-empathy toward others. This would have theoretical and practical significance. Future studies should develop a scale to specifically measure counter-empathy.

Third, although participants were only required to pay attention to the partner’s facial expression, understand the other person's situation, experience the other person's feelings, it is still possible that complicated emotional and cognitive processes, such as the motivation to receive the reward, may be involved in the counter-empathic responses towards the partner. However, disentangle such the motivational processing could be very difficult in counter-empathic responses, as well as in empathic responses. In real-life situations, empathic and counter-empathic responses are both always accomplished with motivation. Thus, a situation that involves no motivation is difficult to achieve. Nevertheless, we expect that researchers can further improve our paradigm to control extraneous variables.

**Funding sources**

This work was supported by the National Natural Science Foundation of China (31771218,31970996), Ministry of Education in China Project of Humanities and Social Sciences (19YJCZH067), Major Program of the National Social Science Foundation of China (14ZDB159), and counselling bases for the Happy Guangzhou project, which received funding from the Guangzhou government.

**CRediT authorship contribution statement**

**Jie Jing:** Conceptualization, Methodology, Software, Validation, Formal analysis, Investigation, Data curation, Writing, Visualization. **Fan Min:** Methodology, Software, Formal analysis, Investigation, Resources, Data curation. **Yang Yong:** Methodology, Investigation, Resources. **Luo Pinchao:** Methodology, Investigation. **Wang Yijing:** Investigation, Resources. **Li Junjiao:** Software. **Chen Wei:** Software. **Zhuang Mengdi:** Methodology. **Zheng Xifu:** Conceptualization, Supervision, Project administration, Funding acquisition.

**Data Availability Statement**

The data that support the findings of this study are available on request from the corresponding author. The data are not publicly available due to privacy or ethical restrictions.

**Conflicts of interest**

None.

**References**

Aderman, D., & Unterberger, G. L. (1977). Contrast empathy and observer modeling behavior. *Journal of Personality, 45*, 267–280.

Adolphs, R., Damasio, H., Tranel, D., Cooper, G. E., & Damasio, A. R. (2000). A role for somatosensory cortices in the visual recognition of emotion as revealed by three-dimensional lesion mapping. *The Journal of Neuroscience, 20*(7), 2683–2690.

Balconi, M., Pozzoli, U. (2007). Event-related oscillation (EROs) and event-related potentials (ERPs) comparison in facial expression recognition. *Journal of Neuropsychology,1*,283–294.

Balconi, M., Pozzoli, U. (2009). Arousal effect on emotional face comprehension. Frequency band changes in different time intervals. *Physiology & Behavior, 97*(3–4),455–462.

Boecker, L., Likowski, K. U., Pauli, P., & Weyers, P. (2015). The face of schadenfreude: differentiation of joy and schadenfreude by electromyography. *Cognition & Emotion, 29*(6), 1117–1125. doi:10.1080/02699931.2014.966063

Borich, M. R., Brodie, S. M., Gray, W. A., Ionta, S., & Boyd, L. A. (2015). Understanding the role of the primary somatosensory cortex: opportunities for rehabilitation. *Neuropsychologia, 79*(Pt B), 246–255.

Breiter, H. C., Aharon, I., Kahneman, D., Dale, A. M., & Shizgal, P. (2001). Functional imaging of neural responses to expectancy and experience of monetary gains and losses. *Neuron, 30*(2), 619–639.

Buckner, R. L., Andrews-Hanna, J. R., & Schacter, D. L. (2008). The brain’s default network: anatomy, function, and relevance to disease. *Annals of the New York Academy of Sciences, 1124*, 1–38.

Carrington, S. J., & Bailey, A. J. (2009). Are there theory of mind regions in the brain? A review of the neuroimaging literature. *Human Brain Mapping, 30*(8), 2313–2335.

Carr, L., Iacoboni, M., Dubeau, M. C., Mazziotta, J. C., & Lenzi, G. L. (2003). Neural mechanisms of empathy in humans: a relay from neural systems for imitation to limbic areas. *Proceedings of the National Academy of Sciences of the United States of America, 100*(9), 5497–5502. doi:10.1073/pnas.0935845100

Chambers, C. D., Payne, J. M., Stokes, M. G., & Mattingley, J. B. (2004). Fast and slow parietal pathways mediate spatial attention. *Nature Neuroscience,* 7, 217– 218.

Chiao, J. Y., & Mathur, V. A. (2010). Intergroup empathy: how does race affect empathic neural responses? *Current Biology, 20*(11), R478–480.

Critchley, H. D., Daly, E., Phillips, M. L., Brammer, M., Bullmore, E. T., Williams, S. C., Amelsvoort, T. V., Robertson, D., David, A.,& Murphy, D. (2000). Explicit and implicit neural mechanisms for processing of social information from facial expressions: a functional magnetic resonance imaging study. *Human Brain Mapping, 9*(2), 93–105.

Decety, J., Echols, S., & Correll, J. (2010). The blame game: the effect of responsibility and social stigma on empathy for pain. *Journal of Cognitive Neuroscience, 22*(5), 985–997.

Decety, J., & Grezes, J. (2006). The power of simulation: imagining one's own and other's behavior. *Brain Research, 1079*(1), 4–14.

de Greck, M., Scheidt, L., Bölter, A. F., Frommer, J., Ulrich, C., Stockum, E., Enzi, B., Tempelmann, C., Hoffmann, T., Han S., Northoff, G. (2012). Altered brain activity during emotional empathy in somatoform disorder. *Human Brain Mapping, 33*(11), 2666-2685. doi:10.1002/hbm.21392

de Greck, M., Shi, Z., Wang, G., Zuo, X., Yang, X., Wang, X., Northoff, G., Han, S. (2012). Culture modulates brain activity during empathy with anger. *Neuroimage, 59*(3), 2871–2882. doi:10.1016/j.neuroimage.2011.09.052

Der Werf, Y. D., Tisserand, D. J., Visser, P. J., Hofman, P., Vuurman, E. F., Uylings, H. B., & Jolles, J. (2001). Thalamic volume predicts performance on tests of cognitive speed and decreases in healthy aging. *Cognitive Brain Research, 11*(3), 377–385.

de Waal, F.B.(2007). Putting the altruism back into altruism: the evolution of empathy. *Annual Review of Psychology, 59*,1–22.

Dvash, J., Gilam, G., Ben-Ze'ev, A., Hendler, T., & Shamay-Tsoory, S. G. (2010). The envious brain: the neural basis of social comparison. *Human Brain Mapping, 31*(11), 1741–1750.

Englis, B. G., Vaughan, E. B., & Lanzetta, J. T. (1982). Conditioning of counter-empathic emotional responses. *Journal of Experimental Social Psychology, 18,* 375–391.

Fan, M., Jie. J., Luo. P., Pang. Y., Xu. D.,Yu. G., Zhao. S., Chen. W., & Zheng, X.(2021) Social exclusion down-regulates pain empathy at the late stage of empathic responses: electrophysiological evidence. *Frontiers in Human Neuroscience*, 15:634714. doi: 10.3389/fnhum.2021.634714

Feng, C., Li, Z., Feng, X., Wang, L., Tian, T., & Luo, Y. J. (2016). Social hierarchy modulates neural responses of empathy for pain. *Social Cognitive and Affective Neuroscience, 11*(3), 485–495.

Fox, E., Russo, R., Bowles, R., & Dutton, K. (2001). Do threatening stimuli draw or hold visual attention in subclinical anxiety? *Journal of Experimental Psychology: General, 130*(4), 681–700.

Friston, K., Worsley, K., Frackowiak, R., Mazziotta, J., & Evans, A. (1994). Assessing the significance of focal activations using their spatial extent. *Human Brain Mapping, 1*, 210–220.

Gallagher, H.L., Happé, F., Brunswick, N., Fletcher, P.C., Frith, U., & Frith, C.D.(2000).Reading the mind in cartoons and stories: An fMRI study of 'theory of mind' in verbal and nonverbal tasks. *Neuropsychologia,38,*11– 21.

Gao, X., Yu, H., Saez, I., Blue, P. R., Zhu, L., Hsu, M., & Zhou, X. (2018). Distinguishing neural correlates of context-dependent advantageous- and disadvantageous-inequity aversion. *Proceedings of the National Academy of Sciences of the United States of America, 115*(33), E7680–E7689.

Gottfried, J. A., Odoherty, J. P., & Dolan, R. J. (2003). Encoding predictive reward value in human amygdala and orbitofrontal cortex. *Science, 301*(5636), 1104–1107.

Goubert, L., Craig, K. D., Vervoort, T., Morley, S., Sullivan, M. J., Williams, A. C. D. C., Cano, A., & Crombez, G. (2005). Facing others in pain: the effects of empathy. *Pain, 118*(3), 285–288.

Gross, J. J. (1998). The emerging field of emotion regulation: an integrative review. Review of General Psychology, 2(3), 271–299.Han, S. (2018). Neurocognitive basis of racial ingroup bias in empathy. *Trends in Cognitive Sciences, 22*(5), 400–421.

Han, S. (2018). Neurocognitive basis of racial ingroup bias in empathy. *Trends in Cognitive Sciences, 22*(5), 400–421.

Haxby, J. V., Hoffman, E. A., & Gobbini, M. I. (2002). Human neural systems for face recognition and social communication. *Biological Psychiatry, 51*(1), 59–67.

Hein, G., Silani, G., Preuschoff, K., Batson, C. D., & Singer, T. (2010). Neural responses to ingroup and outgroup members' suffering predict individual differences in costly helping. *Neuron, 68*(1), 149–160.

Hein, G., & Singer, T. (2008). I feel how you feel but not always: the empathic brain and its modulation. *Current Opinion in Neurobiology, 18*(2), 153–158.

Hooker, C. I., Verosky, S. C., Germine, L. T., Knight, R. T., & D'Esposito, M. (2010). Neural activity during social signal perception correlates with self-reported empathy. *Brain Research, 1308*, 100–113.

Itagaki, S., & Katayama, J. (2008). Self-relevant criteria determine the evaluation of outcomes induced by others. *Neuroreport, 19*(3), 383–387.

Japee, S., Holiday, K., Satyshur, M. D., Mukai, I., & Ungerleider, L. G. (2015). A role of right middle frontal gyrus in reorienting of attention: a case study. *Frontiers in Systems Neuroscience, 9*, 23.

Jie, J., Luo, P., Zhuang, M., Fan, M., Wang, Y., Yang, Y., & Zheng, X. (2019). Gender differences in empathic responses to others’ economic payoffs: An event-related potentials study. *Experimental Brain Research, 237*(5), 1347–1359. doi: 10.1007/s00221-019-05518-x. Epub 2019 Mar 15.

Jie, J., Luo, P., Zhuang, M., Li, B., Pang, Y., Li, J., & Zheng, X. (2019). Self-interest induces counter-empathy at the late stage of empathic responses to others’ economic payoffs. *Frontiers in Psychology, 10*, 372. doi: 10.3389/fpsyg.2019.00372

Kandylaki, K. D., Nagels, A., Tune, S., Wiese, R., Bornkessel-Schlesewsky, I., & Kircher, T. (2015). Processing of false belief passages during natural story comprehension: An fMRI study. *Human Brain Mapping, 36*(11), 4231–4246.

Kanwisher, N., Mcdermott, J. H., & Chun, M. M. (1997). The fusiform face area: a module in human extrastriate cortex specialized for face perception. *The Journal of Neuroscience, 17*(11), 4302–4311.

Kim, P., Evans, G. W., Angstadt, M., Ho, S. S., Sripada, C., Swain, J. E., Liberzonc, I., & Phan, K. L. (2013). Effects of childhood poverty and chronic stress on emotion regulatory brain function in adulthood. *Proceedings of the National Academy of Sciences of the United States of America,* *110*(46), 18442–18447.

Kohn, N., Eickhoff, S. B., Scheller, M., Laird, A. R., Fox, P. T., & Habel, U. (2014). Neural network of cognitive emotion regulation--an ALE meta-analysis and MACM analysis. *Neuroimage, 87*, 345–355.

Krautheim, J. T., Dannlowski, U., Steines, M., Neziroğlu, G., Acosta, H., Sommer, J., Straube, B., & Kircher, T. (2019). Intergroup empathy: Enhanced neural resonance for ingroup facial emotion in a shared neural production-perception network. *Neuroimage, 194*, 182–190. doi:10.1016/j.neuroimage.2019.03.048

Lakens, D., & Caldwell, A. R.(2021). Simulation-Based Power Analysis for Factorial Analysis of Variance Designs. *Advances in Methods and Practices in Psychological Science, 4*(1), 1–14.

Lamm, C., Batson, C. D., & Decety, J. (2007). The Neural Substrate of Human Empathy Effects of Perspective-Taking and Cognitive Appraisal. *Journal of Cognitive Neuroscience, 19*(1), 42–58.

Lang, S., Yu, T., Markl, A., Muller, F., & Kotchoubey, B. (2011). Hearing others' pain: neural activity related to empathy. *Cognitive, Affective, & Behavioral Neuroscience, 11*(3), 386–395.

Li, Y., Sescousse, G., Amiez, C., & Dreher, J. C. (2015). Local morphology predicts functional organization of experienced value signals in the human orbitofrontal cortex. *The Journal of Neuroscience, 35*(4), 1648–1658.

Luo, P., Pang, Y., Li, B., Jie, J., Zhuang, M., Yang, S., & Zheng, X. (2018). Competitive intensity modulates the pain empathy response: an event-related potentials study. *Frontiers in psychology, 9*, 1854.

Luo, P., Zhuang, M., Jie, J., Wu, X., & Zheng, X. (2018). State anxiety down-regulates empathic responses: electrophysiological evidence. *Frontiers in human neuroscience, 12*, 502.

Mainen, Z. F., & Kepecs, A. (2009). Neural representation of behavioral outcomes in the orbitofrontal cortex. *Current Opinion in Neurobiology, 19*(1), 84–91.

Mendes, N., Steinbeis, N., Bueno-Guerra, N., Call, J., & Singer, T. (2018). Preschool children and chimpanzees incur costs to watch punishment of antisocial others. *Nature Human Behaviour, 2*(1), 45–51.

Nagahama, Y., Okada, T., Katsumi, Y., Hayashi, T., Yamauchi, H., Sawamoto, N., Toma, K., Nakamura, K., Hanakawa, T., Konishi, J., Fukuyama, H., & Shibasaki, H. (1999). Transient neural activity in the medial superior frontal gyrus and precuneus time locked with attention shift between object features. *Neuroimage, 10*(2), 193–199.

Pardini, D. A., Lochman, J. E., & Frick, P. J. (2003). Callous/unemotional traits and social-cognitive processes in adjudicated youths. *Journal of the American Academy of Child and Adolescent Psychiatry, 42*(3), 364–371.

Perry, D., Hendler, T., & Shamay-Tsoory, S. G. (2012). Can we share the joy of others? Empathic neural responses to distress vs joy. *Social Cognitive and Affective Neuroscience, 7*(8), 909–916.

Pfeifer, J. H., Iacoboni, M., Mazziotta, J. C., & Dapretto, M. (2008). Mirroring others' emotions relates to empathy and interpersonal competence in children. *Neuroimage, 39*(4), 2076–2085.

Preston, S. D., & De Waal, F. B. (2002). Empathy: its ultimate and proximate bases. *Behavioral and Brain Sciences, 25*(1), 1–72.

Rohr, C. S., Villringer, A., Solms-Baruth, C., van der Meer, E., Margulies, D. S., & Okon-Singer, H. (2016). The neural networks of subjectively evaluated emotional conflicts. *Human Brain Mapping, 37*(6), 2234–2246.

Rolls, E. T. (2000). The orbitofrontal cortex and reward. *Cerebral Cortex, 10*(3), 284–294.

Schulte-Rüther, M., Markowitsch, H. J., Shah, N. J., Fink, G. R., & Piefke, M. (2008). Gender differences in brain networks supporting empathy. *Neuroimage, 42*(1), 393–403. doi:10.1016/j.neuroimage.2008.04.180

Seehausen, M., Kazzer, P., Bajbouj, M., Heekeren, H. R., Jacobs, A. M., Klann-Delius, G., Menninghaus, W., & Prehn, K. (2014). Talking about social conflict in the MRI scanner: neural correlates of being empathized with. *Neuroimage, 84*, 951–961.

Seghier, M. L. (2013). The angular gyrus: multiple functions and multiple subdivisions. *Neuroscientist, 19*(1), 43–61.

Shamay-Tsoory, S. G., Aharon-Peretz, J., & Perry, D. (2009). Two systems for empathy: a double dissociation between emotional and cognitive empathy in inferior frontal gyrus versus ventromedial prefrontal lesions. *Brain, 132*(Pt 3), 617–627.

Singer, T., Seymour, B., O'Doherty, J., Kaube, H., Dolan, R. J., & Frith, C. D. (2004). Empathy for pain involves the affective but not sensory components of pain. Science, 303(5661), 1157-1162.

Singer, T., Seymour, B., O'Doherty, J. P., Stephan, K. E., Dolan, R. J., & Frith, C. D. (2006). Empathic neural responses are modulated by the perceived fairness of others. *Nature, 439*(7075), 466–469.

Stotland, E., Sherman, S., & Shaver, K. (1971). *Empathy and birth order: Some experimental explorations*. Lincoln: University of Nebraska Press.

Takahashi, H., Kato, M., Matsuura, M., Mobbs, D., Suhara, T., & Okubo, Y. (2009). When your gain is my pain and your pain is my gain: neural correlates of envy and schadenfreude. *Science, 323*(5916), 937–939.

Tian, L., Jiang, S., & Huebner, E. S. (2019). The big two personality traits and adolescents’ complete mental health: The mediation role of perceived school stress. *School Psychology, 34*(1), 32–42.

Tversky, A., & Kahneman, D. (1991). Loss aversion in riskless choice: a reference-dependent model. *Quarterly Journal of Economics, 106*(4), 1039–1061.

Tzourio-Mazoyer, N., Landeau, B., Papathanassiou, D., Crivello, F., Etard, O., Delcroix, N., Mazoyer, B., & Joliot, M. (2002). Automated anatomical labeling of activations in SPM using a macroscopic anatomical parcellation of the MNI MRI single-subject brain. *Neuroimage, 15*(1), 273–289.

Völlm, B. A., Taylor, A. N., Richardson, P., Corcoran, R., Stirling, J., McKie, S., Deakin, J. F. W., & Elliott, R. (2006). Neuronal correlates of theory of mind and empathy: a functional magnetic resonance imaging study in a nonverbal task. *Neuroimage, 29*(1), 90–98.

Vuilleumier, P., Armony, J. L., Driver, J., & Dolan, R. J. (2003). Distinct spatial frequency sensitivities for processing faces and emotional expressions. *Nature Neuroscience, 6*(6), 624–631.

Wicker, B., Keysers, C., Plailly, J., Royet, J. P., Gallese, V., Rizzolatti, G. (2003). Both of us disgusted in My Insula: The common neural basis of seeing and feeling disgust. *Neuron,40*(3), 655–664.

Xia, M., Wang, J., & He, Y. (2013). BrainNet viewer: a network visualization tool for human brain connectomics. *PLoS One, 8*(7), e68910.

Yamada, M., Lamm, C., & Decety, J. (2011). Pleasing frowns, disappointing smiles: an ERP investigation of counterempathy. *Emotion, 11*(6), 1336–1345.

Yan, C. G., Wang, X. D., Zuo, X. N., & Zang, Y. F. (2016). DPABI: data processing & analysis for (resting-state) brain imaging. *Neuroinformatics, 14*, 339–351.

Yiend, J., & Mathews, A. (2001). Anxiety and attention to threatening pictures. *Quarterly Journal of Experimental Psychology, 54*(3), 665–681.

**Figure Legends**

**Figure 1.** Example of the experimental design. At the beginning of each trial, a fixation point was displayed at the center of the screen for 500 –1500 ms. After the fixation point, a 3000-ms flag-matching game was presented. The task result was then presented for 2000 ms, followed by a 4000-ms partner’s facial expression feedback. The participants then completed a 6000-ms rating scale of the subjective pleasantness. The red box represents the stimulation interface for the brain data analysis.

**Figure 2.** Comparison of subjective feeling ratings between the empathy and counter-empathy conditions (* *p* < 0.05; ** *p* < 0.01; *** *p* < 0.001)

**Figure 3.** Comparison of subjective feeling ratings for different distributions to participants’ partners (* *p* < 0.05; ** *p* < 0.01; *** *p* < 0.001)

**Figure 4.** Brain activation in the empathy experiment (*p* < 0.001 at the voxel level, *p* < 0.05 FWE-corrected at the cluster level)

**Figure 5.** Brain activation in the counter-empathy task (*p* < 0.001 at the voxel level, *p* < 0.05 FWE-corrected at the cluster level)

**Figure 6.** Main contrast results for the C_DI distribution (no benefit) to others. (A) Brain activation for empathy > counter-empathy; (B) Brain activation for counter-empathy > empathy (*p* < 0.001 at the voxel level, *p* < 0.05 FWE-corrected at the cluster level)

**Figure 7.** Main contrast results for I_DI distribution (10 yuan deducted) to others. (A) Brain activation for empathy > counter-empathy; (B) Brain activation for counter-empathy > empathy (*p* < 0.001 at the voxel level, *p* < 0.05 FWE-corrected at the cluster level)

**Figure 8.** Brain activation for counter-empathy > empathy in the C_AI distribution (winning 10 yuan) to the partner (*p* < 0.001 at the voxel level, *p* < 0.05 FWE-corrected at the cluster level)

**Figure 9.** Brain activation for empathy > counter-empathy in I_AI distribution (no loss) to partner (*p* < 0.001 at the voxel level, *p* < 0.05 FWE-corrected at the cluster level)

**Figure 10.** Brain activation in the DI distribution > AI distribution to others during incorrect flag-matching (*p* < 0.001 at the voxel level, *p* < 0.05 FWE-corrected at the cluster level)

**Figure 11.** I_DI distribution > C_DI distribution to partner activation contrasts (*p* < 0.001 at the voxel level, *p* < 0.05 FWE-corrected at the cluster level)

**Figure 12.** Process model of counter-empathy

(A) Process model of counter-empathy without prejudice; (B) Process model of counter-empathy with prejudice

**2. Material and Methods**

***2.1.*** ***Participants***

We performed power and sample size estimations using Superpower(Lakens & Caldwell, 2021) in the online Shiny apps (see https://arcstats.io/shiny/anova-power/). We set groups to 2w*2w*3w for the simulation, n = 20(20 participants), a correlation among repeated measures of 0.5, and an alpha of 0.05. In this case, we simulate data with means of subjective pleasure ratings in the empathy_R_DI, empathy_R_FA, empathy_R_AI, empathy_W_DI, empathy_W_FA, empathy_W_AI, counter-empathy_R_DI, counter-empathy_R_FA, counter-empathy_R_AI, counter-empathy_W_DI, counter-empathy_W_FA, and counter-empathy_W_AI conditions are 3,5,7,2,4,6,7,6,4,6,3,3, respectively, and set the sd to 1. A Greenhouse-Geisser sphericity correction were applied for correction of violations of sphericity and a Holm-Bonferroni correction were calculated for multiple comparisons. The number of simulations is 10000. Simulations showed that we have 92.44% power for main effect of condition, effect size = 0.42, 100% power for main effect of feedback, effect size = 0.92, 99.22% power for the main effect of fairness, effect size = 0.42, 92.58% power for condition*feedback interaction, effect size = 0.42, 100% power for condition*fairness interaction, effect size = 0.96, 99.31% power for feedback*fairness interaction, effect size = 0.42, 99.41% power for condition*feedback* fairness interaction, effect size = 0.43.
